# Out FOXing Parkinson Disease: Where Development Meets Neurodegeneration

**DOI:** 10.1371/journal.pbio.0050334

**Published:** 2007-12-18

**Authors:** Eric M Wexler, Daniel H Geschwind

## Abstract

The central survival role of FOX proteins may allow a unified view of the genetic and environmental factors that cause Parkinson disease.

Parkinson disease (PD) is the most common neurodegenerative movement disorder in humans, but its cause in the majority of patients is unknown. Fewer than 10% of cases appear to be inherited, and only rarely are the known Mendelian mutations identified in patients with PD. In addition to genetic factors, environmental toxins—especially those affecting mitochondrial function—have also been implicated [1,2], demonstrating a clear role for gene–environment interactions in most cases of sporadic PD. Patients afflicted with PD experience slow, selective, often asymmetric loss of dopaminergic (DA) neurons projecting from the substantia nigra (SN) to the striatum, but not of DA neurons located elsewhere in the brain (e.g., ventral tegmental area and retina). Once more than 80%–90% of DA neurons in the SN are lost, patients suffer the characteristic symptoms of tremor, slow movements (bradykinesia), rigidity, and postural instability.

To study PD, researchers have used two basic types of animal models: (1) acute lesioning of DA neurons/tracts, either by surgery or toxic insult, and (2) genetic models that rely on expression of one of the rare, dominant, and highly penetrant mutations that cause PD or knockout of a similar, recessively inherited allele (e.g., α-synuclein, Parkin, and leucine-rich repeat kinase-2 [LRRK2]). Although these models have led to huge advances in our understanding of PD—implicating mitochondrial dysfunction, oxidative damage, and protein handling systems in pathogenesis—they typically either fail to recapitulate the gradual nature of this degenerative disease or lack the defining pathology (e.g., Parkin and α-synuclein transgenic mice fail to show progressive nigrostriatal degeneration, although rotenone-treated mice do show Lewy bodies (intracytoplasmic inclusions that are composed primarily of α-synuclein and ubiqutin)) [3,4]. Because no single animal model is likely to be sufficient for the study of any particular human neurodegenerative disease, new models are needed to accelerate our ability to devise and test improved disease-altering therapies.

It is in this regard that a new paper in *PLoS Biology* by Kittappa et al. [5] warrants special attention. They demonstrate that knockout of a transcription factor FoxA2, which is critical for DA neuron specification and survival in mice causes a late-onset, asymmetric degenerative condition affecting motor systems in a manner very similar to PD. This model is entirely distinct in its basic mechanism from the aforementioned models, because it involves partial loss of a transcription factor that is critical for DA neuron cell fate. One of the most striking features of the majority of adult-onset neurodegenerative diseases is their regional or cell-type specificity in humans. Yet, many of the known causative mutations reside in proteins that are widely expressed throughout the central nervous system, such as synuclein, LRK2, and Parkin in PD, beta amyloid and presenilin-1 (PS1) in Alzheimer disease, and tau and progranulin in frontotemporal dementia. The current study in mice, and previous work in humans and other model organisms, highlight the potential role that neurodevelopmental factors may play in the cell-type–specific or regional neuronal vulnerability seen in neurodegenerative disease [6,7] As this work by Kittappa and colleagues demonstrates, understanding early developmental processes such as cell-type specification and survival may have major implications for aging-related diseases.

## Development of Nigral DA Neurons

Dopamine neurons are initially generated in a permissive region at the embryonic midbrain–hindbrain border (isthmus). This region is formed by intersecting gradients of several molecules: fibroblast growth factor-8 (FGF8), which is produced by cells at the isthmus, secreted sonic hedgehog (Shh) from the ventral neural tube and floorplate, and Wnt-1 and Wnt5a from the midbrain and rostral diencephalons.. These factors induce a proneuronal program in dividing neuroepithelial precursors that specifically favors DA differentiation and concomitantly suppresses differentiation in non-DA neurons (reviewed in[8]). The anatomic distribution of these factors is illustrated in Figure 1.

**Figure 1:**
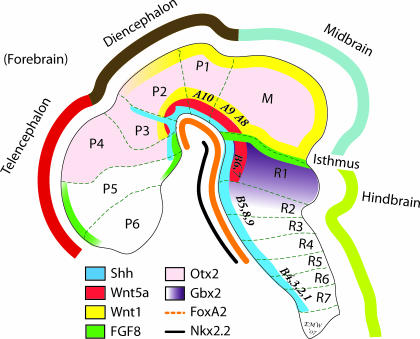
Factors influencing monoaminergic cell development in regions of FoxA2 expression Schematic of brain, approximately E8-11. At earlier stages (not shown), Lmx1b, Wnt1, Grg4, Otx2, and Gbx2 set up complex parallel feedback loops that establish the FGF8 secreting isthmus organizer at the midbrain–hindbrain boundary. Shown here, at later stages, Otx2 represses Nkx2.2 expression in the mesodiencephalon. Shh, which is produced by cells of the floor plate and notochord, induces FoxA2 expression in the rhombencephalon (hindbrain), mesencephalon (midbrain), and caudal prosencephalon (dielcephalon). DA neurons are formed in those regions of FoxA2 expression where Nkx2.2 is repressed. Nine groups of dopaminergic neurons are formed in the forebrain and midbrain. Only groups A8–10 are shown. A8/9 cells give rise to the SN, whereas A10 cells eventually comprise the ventral tegmental area (VTN). Prosomeres, P1–P6; rhombmeres, R1–R7; serotonergic cells, B1–B9; DA cells, A8–A10. Figure is adapted from [8,19–21]

Two recent studies show that Shh and FoxA1/2 are required for differentiation of both DA neurons and the serotonergic (5HT) neurons [9,10]. Interestingly, these studies suggest that both Shh and FoxA2 are able to induce the other's expression. Kittappa et al., unravel some of the complexity of this interaction. They demonstrate that Shh-induced FoxA2 expression is responsible for driving the DA differentiation program. However, FoxA2 also induces continued Shh expression, which can drive proliferation of uncommitted progenitors, promoting their survival once differentiated [11]**.** Defining an initiating role for FoxA2 is good news for the production of DA neurons from embryonic stem (ES) cells, but previous studies left open the intriguing question of what role FoxA2 plays in the functioning of adult DA neurons.

## A New Mouse Model of PD That Connects Development with Adult Neurodegeneration

The article by Kittappa et al. [5] highlights two important findings that could change the terrain for investigating the etiology of PD. As mentioned above, the authors clarify the developmental origins of DA neurons and establish a framework of transcription factors that provides a context for the experimental regulation of midbrain neuron differentiation. Second, they report a highly unique age-dependent onset of DA cell loss, which begins asymmetrically in the brain.

Because FoxA2-null mutants die prenatally, Kittappa et al. explored the effects of missing just one copy of FoxA2 (haploinsufficiency) in aging heterozygous FoxA2^+/−^ mice. These mice show no gross histological or behavioral abnormalities throughout most of their life, but when the mice are in late adulthood (18 mo old), the researchers observed that approximately one-third of animals begin to develop progressive muscle rigidity with asymmetric posturing and tremors, not seen in age-matched wild-type littermates. These symptoms, which are suggestive of striatal degeneration, prompted the authors to demonstrate a functional lesion of the dopaminergic system using the standard amphetamine-induced rotation assay. Affected animals showed significant, asymmetric loss of DA neurons in the SN, particularly among retinoic acid–producing neurons, but the animals showed no loss in the ventral tegmental area. In contrast, age-matched FoxA2^+/−^ mice that displayed a normal behavioral phenotype had no attendant loss of DA neurons. Interestingly, affected animals exhibited bilateral activation of astrocytes in multiple midbrain areas, suggesting that some effects of haploinsufficiency may be more diffuse.

Together, these findings demonstrate that the authors have generated a new animal model of PD that captures both the late onset and selectivity of dopaminergic degeneration, including asymmetric motor dysfunction, which is a hallmark of the disorder in humans. However, one cardinal pathologic feature of PD not observed in this model is the presence of Lewy bodies [12]. Although Lewy bodies are still considered a pathological feature of PD, they are not a *sine qua non* for the disease. Many patients, including patients harboring PARK2 (Parkin) mutations, lack extensive Lewy body pathology on autopsy yet exhibit all the clinical features of PD [13,14].

## FoxA2 Presents New Opportunities for PD Research

The present study raises a host of interesting questions. First, why does FoxA2 haploinsufficiency lead to progressive loss of DA neurons and their projections to the striatum? Although detailed studies will be required to properly answer this question, one can hypothesize that loss of FoxA2 may be required for proper target innervation; when these projections recede, target-derived trophic support is lost [15] Evidence supporting this speculation comes from studies showing that FoxA2 targets may include genes involved with target innervation and survival of DA neurons [16,17].

Can these exciting findings be extrapolated to better our understanding of human PD or prove useful to developing ways of treating this disease? The fidelity with which this animal model seems to parallel several key aspects of PD in humans tempts one to speculate that some forms of PD may arise through dysfunction of FoxA2. Thus, FoxA2 and its targets are candidates for genetic association studies in PD in humans. Additionally, given the chronicity and late onset observed in the heterozygous mice, as well as the incomplete penetrance at 18 months, these mice provide an exciting opportunity to investigate gene-gene and gene-environment interactions that may affect DA neuron survival in vivo.

Based on these findings, one may also propose that overexpression of FoxA2 could be used to drive DA differentiation of implanted neural stem cells for use in neural repair paradigms in humans with PD. It will also be worthwhile to explore whether increasing FoxA2 protein dosage, by vector-mediated overexpression of FoxA2, is neuroprotective against other genetic or environmental lesions of the nigral DA neuron population. At the same time, we need to recognize that these proposals rely on the assumption that FoxA2 will have the same subset of critical targets in human tissue as it does in mouse. Fraenkel and colleagues [18] analyzed the conservation of FoxA2 binding in mouse versus human hepatocytes. Surprisingly, they found that while FoxA2 bound nearly 600 mouse genes, only 11% of these were also bound in humans. Since promoter binding is state dependent and binding does not necessarily entail transcriptional activity, further study will be needed to understand the appropriate parallels between mouse and human FoxA2 in the DA cells of relevance to PD. In spite of these uncertainties, this new paper provides an entirely fresh perspective on DA neuron survival and a powerful new animal model in which to test gene–environment interactions and new therapeutic approaches in PD.

## Abbreviations

DA: dopaminergic
PD: Parkinson disesase
Shh: Sonic hedgehog
SN: substantia nigra
